# Supplementary material for: Imaging the Binding Between Dasatinib and Its Target Protein in Living Cells Using an SLP Tag System on Intracellular Compartments
Source: Int J Mol Sci. 2025 Jun 13;26(12):5705. doi: 10.3390/ijms26125705 (PMC12193096; doi:10.3390/ijms26125705)
Supplement: Supplementary file 1 [file ijms-26-05705-s001.zip › ijms-3668573-supplementary.pdf]

Supplementary Materials

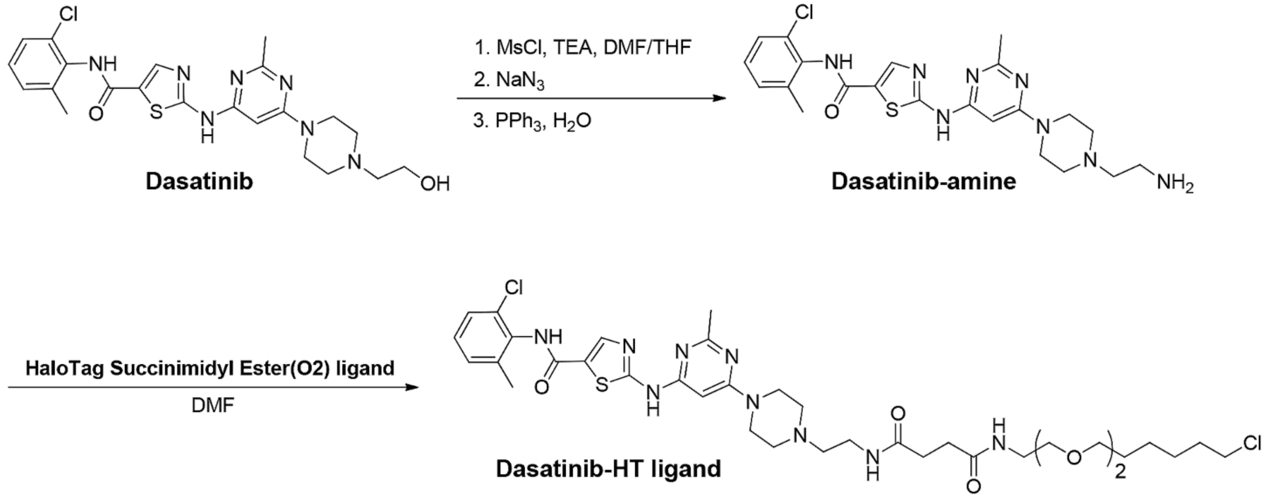

Figure S1. Synthetic scheme of dasatinib-HT ligand.

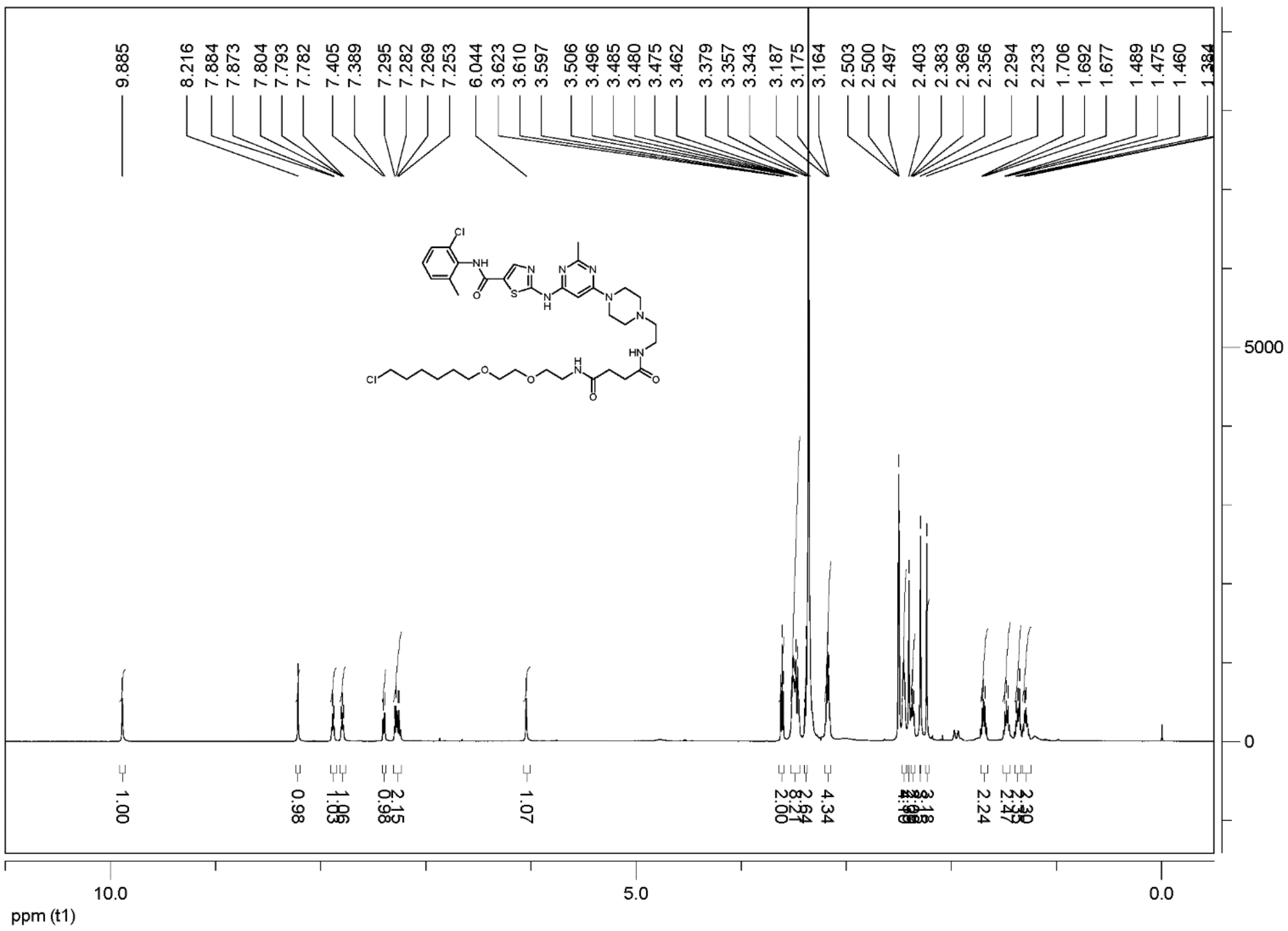

^{1}H NMR (500 MHz, DMSO-d_6) δ 9.88 (br s, 1H), 8.21(br s, 1H), 7.88 (t, J = 5.5 Hz, 1H), 7.79 (t, J = 5.5 Hz, 1H), 7.39 (d, J = 8.0 Hz, 1H), 7.27 (m, 2H), 6.04 (s, 1H), 3.61 (t, J = 6.5 Hz, 2 H), 3.50-3.45 (m, 8H), 3.35 (m, 2H), 3.17 (m, 4H), 2.45 (m, 4H), 2.40 (s, 2H), 2.36 (t, J = 6.5 Hz, 2H), 2.29 (s, 3H), 2.23 (s, 3H), 1.69 (m, 2H), 1.47 (m, 2H), 1.36 (m, 2H), 1.27 (m, 2H).

Figure S2. ^{1}H NMR spectrum of dasatinib-HT lignad.

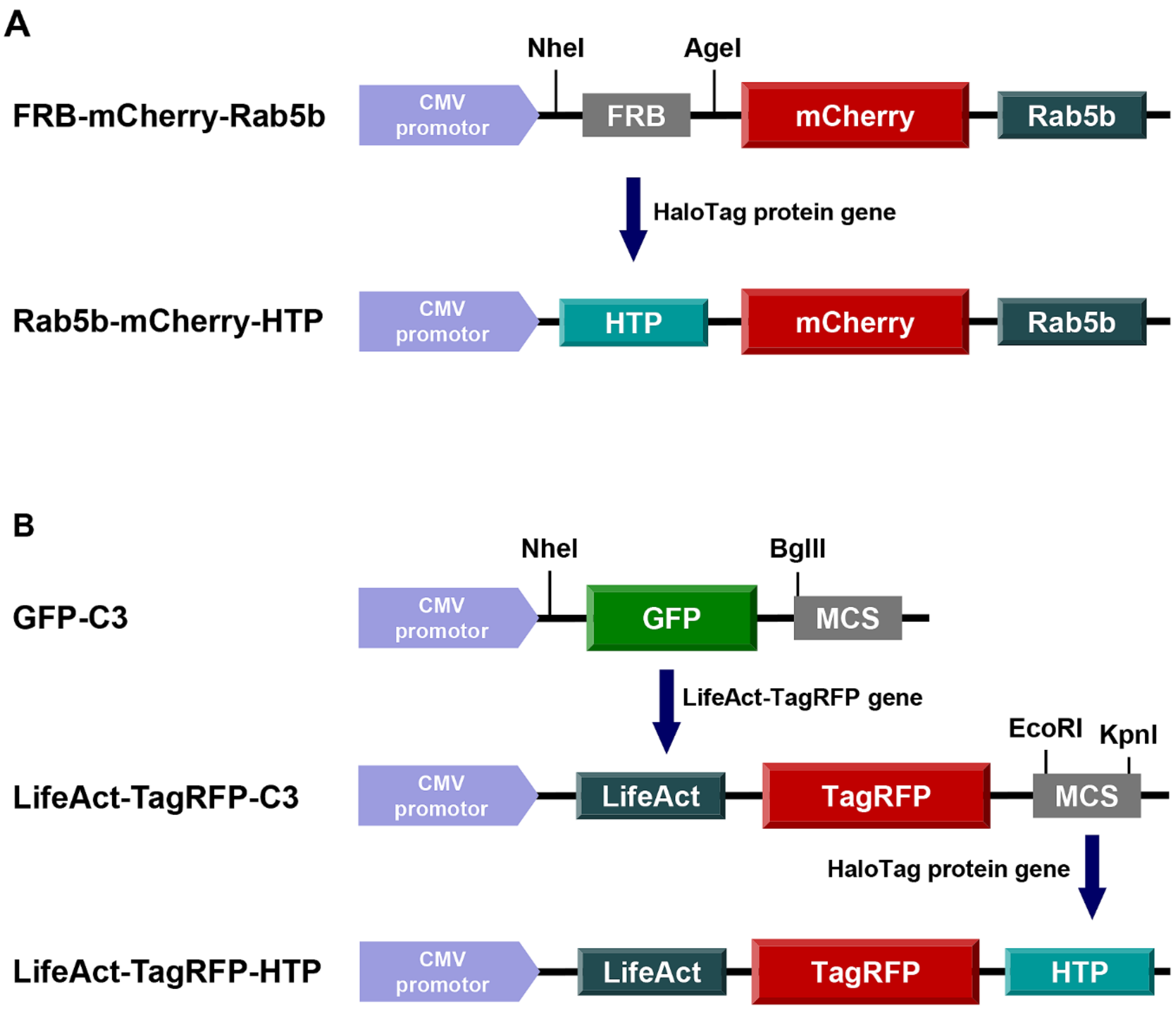

Figure S2. Schematic representation of the HTP expression vectors. (A) Rab5b-mCherry-HTP and (B) LifeAct-TagRFP-HTP.

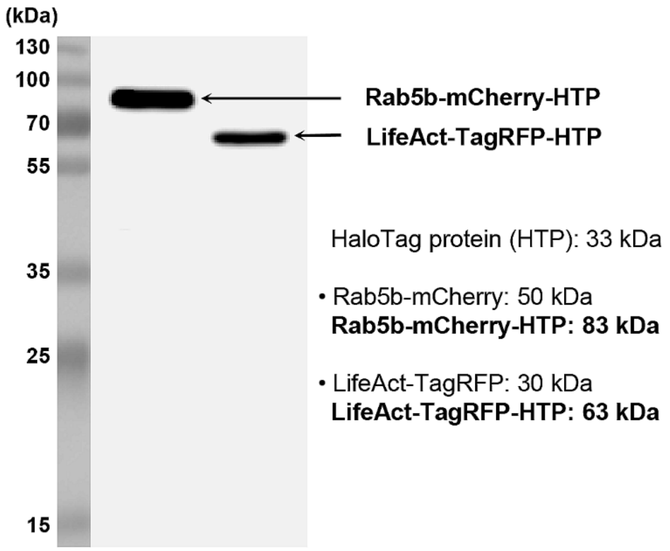

Figure S3. Western blot analysis of lysates from HeLa cells transfected with Rab5b-mCherry-HTP and LifeAct-TagRFP-HTP expression vectors.

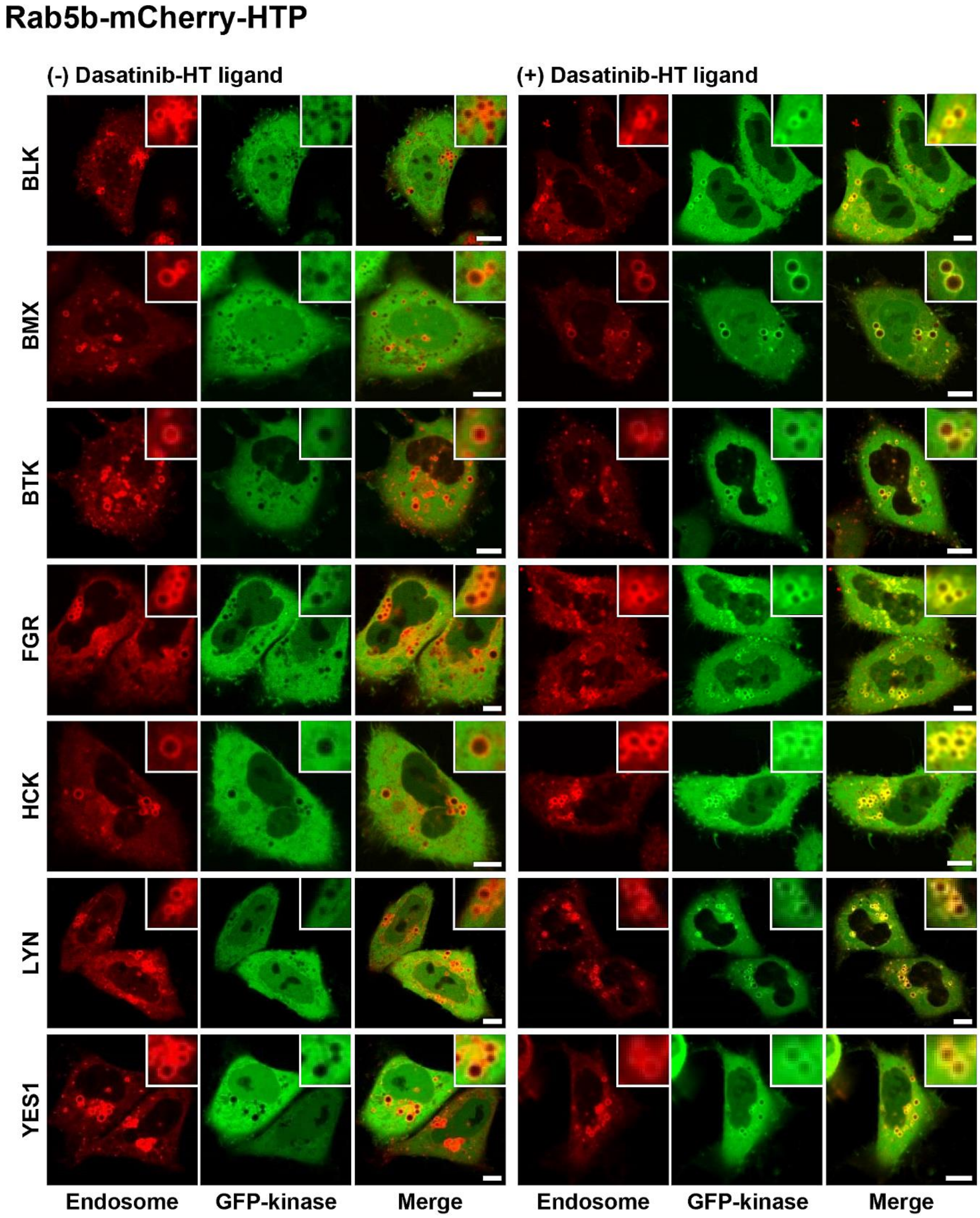

Figure S4. Imaging the binding between dasatinib and its target proteins on the endosome in untreated (left) and dasatinib-HT ligand-treated (right) cells. All scale bars are 10 μm.

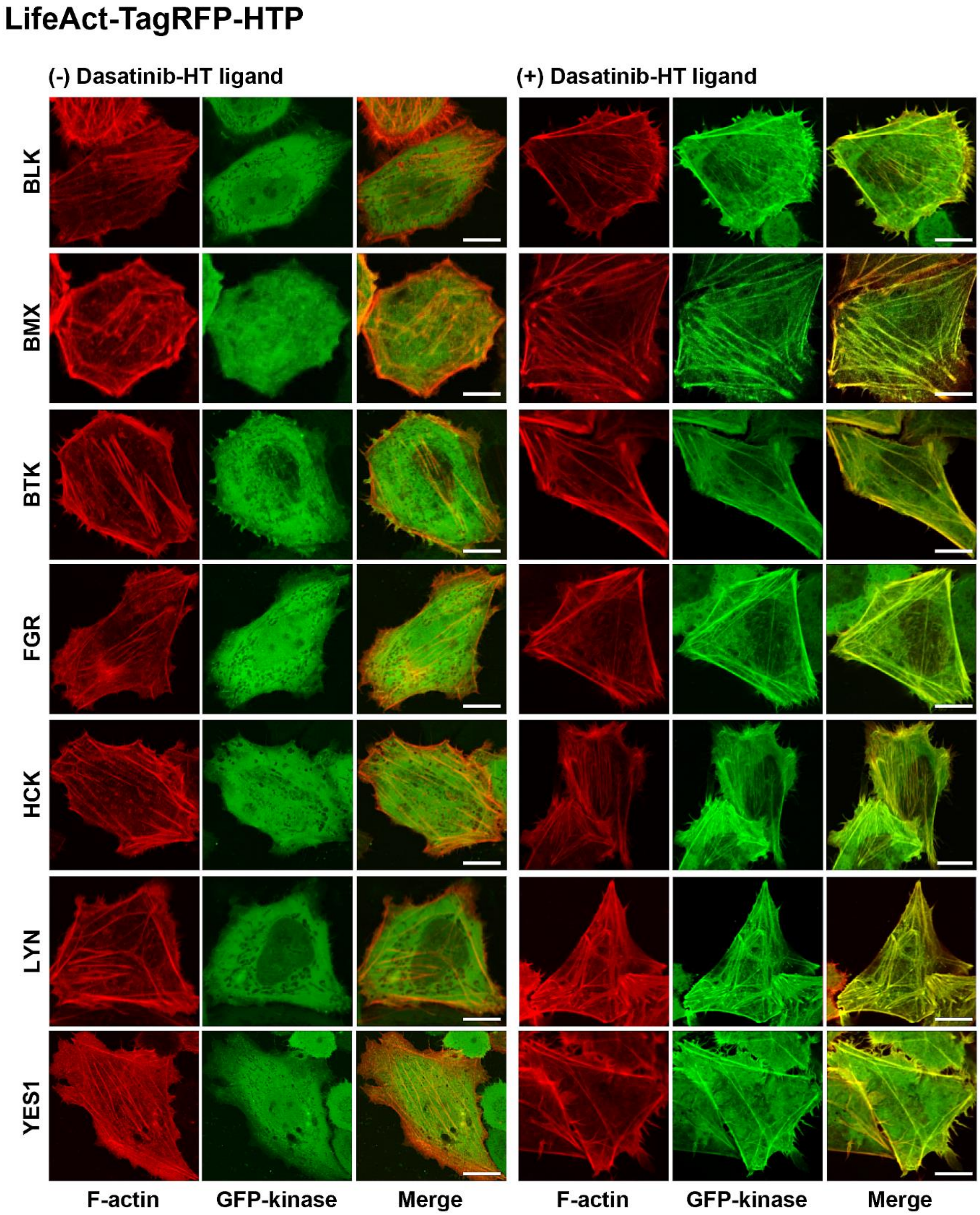

Figure S5. Imaging of the binding between dasatinib and its target proteins on the F-actin in untreated (left) and dasatinib-HT ligand-treated (right) cells. All scale bars are 10 μm.

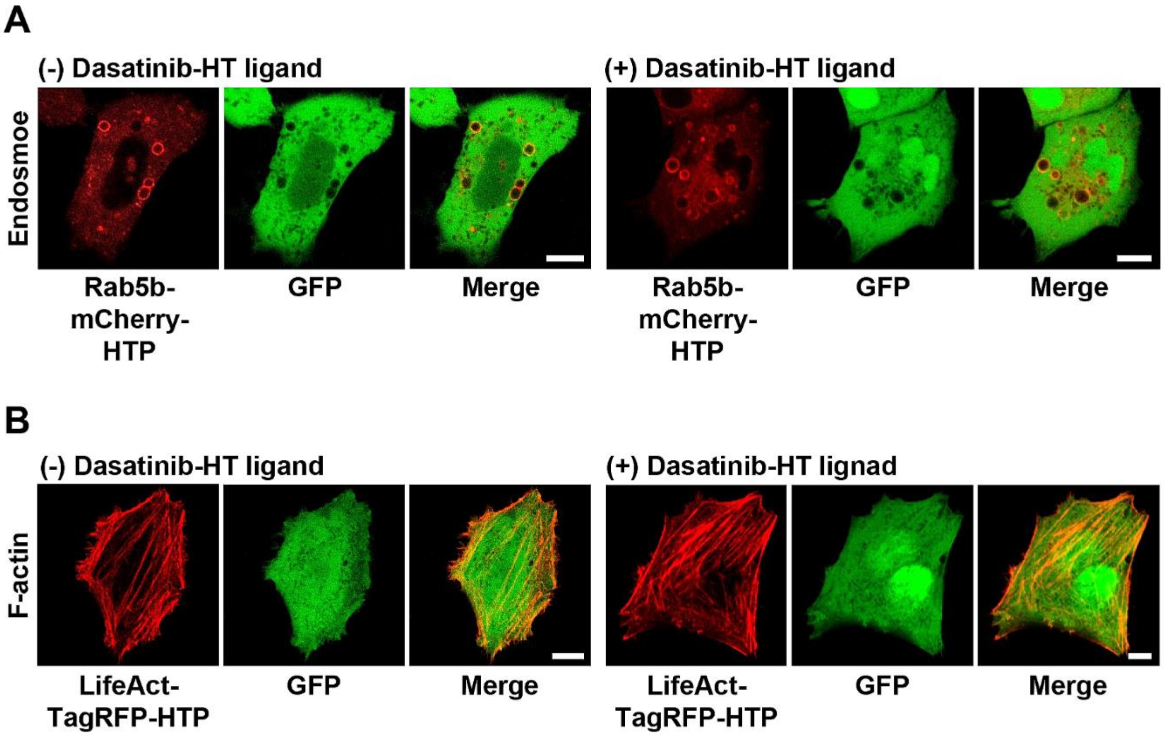

Figure S6. Imaging of cells co-expressing Rab5b−mCherry−HTP (or LifeAct−TagRFP−HTP) and GFP. Dasatinib-HT ligand (1 μM) was nontreated (A) and treated for 1 h (B). All scale bars are 10 μm.

Supporting Table S1. List of primers in this study.

Genes	Primers	vectors	Products
HaloTag (HT)	5'-GACTGCTAGCCCACCATGGGATCCGAAATCGGTACTGG-3' (NheI)<br>5'-GACTACCGGTAGGGCGCCGGCGCCGGCGCCACCGGAAATCTCCAGAGT-3' (AgeI)	FRB-mCherry-Rab5b	HTP-mCherry-Rab5b
LifeAct-TagRFP	5'-CTAGCTAGCCGCCACCATGGGTGTCGCAGATTTGATCAAG-3' (NheI)<br>5'-GAAGATCTGAATTAAGTTTGTGCCCCAGTTTGC-3' (BglII)	EGFP-C3	LifeAct-TagRFP-C3
HaloTag (HT)	5'-GCCGAATTCTATGGGATCCGAAATCGGTACT-3' (EcoRI)<br>5'-CGGGGTACCCCGCTAACCGGAAATCTCCAGAGTAGA-3' (KpnI)	LifeAct-TagRFP-C3	LifeAct-TagRFP-HTP
ABL1[a]	5'-CTACTCGAGCTCATGGTGTCCCCCAACTACGACAAGTG-3' (XhoI)<br>5'-GCCGAATTCGCTGGAACATTGTTTCAAAGGCTT-3' (EcoRI)	EGFP-N1	EGFP-ABL1
BLK	5'-GGACTCGAGCTCATGGGGCTGGTAAGTAGCAA-3' (XhoI)<br>5'-GGAGGATCCTAGGGCTGCAGCTCGTACTG-3' (BamHI)	EGFP-C3	EGFP-BLK
BMX	5'-GCTGAGCTCATGGATACAAAATCTATTCTA-3' (SacI)<br>5'-GCTCCCGGGTCAATGCTTGTCTTTTTCCC-3' (XmaI)	EGFP-C3	EGFP-BMX
BTK	5'-GGTCTCGAGCTCATGGCCGCAGTGATTCTGG-3' (XhoI)<br>5'-GGTGGATCCTAGGATTCTTCATCCATGACAT-3' (BamHI)	EGFP-C3	EGFP-BTK
CSK	5'-GGTGAGCTCATGTCAGCAATACAGGCCGC-3' (SacI)<br>5'-GGTGGATCCTACAGGTGCAGCTCGTGGGTTT-3' (BamHI)	EGFP-C3	EGFP-CSK
FGR	5'-GCTGAATTCTGATGGGCTGTGTGTTCTGCAAG-3' (EcoRI)<br>5'-GCTGGTACCTATGTCTGATCCCCGGGCTG-3' (KpnI)	EGFP-C3	EGFP-FGR
HCK	5'-GCTGAATTCTGATGGGGGGGCGCTCAAGC-3' (EcoRI)<br>5'-GCTGGTACCTATGGCTGCTGTTGGTACTGGC-3' (KpnI)	EGFP-C3	EGFP-HCK
LYN	5'-GCTGAATTCTGATGGGATGTATAAAATCAAAA-3' (EcoRI)<br>5'-GCTGGATCCTAAGGCTGCTGCTGGTATTGCC-3' (BamHI)	EGFP-C3	EGFP- LYN
SRC	5'-GTCCTCGAGCTCATGGGTAGCAACAAGAGCAAG-3' (XhoI)<br>5'-GTCGAATTCCTAGAGGTTCTCCCCGGGC-3' (EcoRI)	EGFP-C3	EGFP-SRC
YES1	5'-GGTGAGCTCATGGGCTGCATTAAAAGTAA-3' (SacI)<br>5'-GGTCCCGGGCTATAAATTTTCTCCTGGCTGGT-3' (XmaI)	EGFP-C3	EGFP- YES1

[a]ABL1 kinase domain (amino acids 247 ~ 517) was used in this study
